# Influence of Welded Pores on Very Long-Life Fatigue Failure of the Electron Beam Welding Joint of TC17 Titanium Alloy

**DOI:** 10.3390/ma12111825

**Published:** 2019-06-05

**Authors:** Fulin Liu, Hong Zhang, Hanqing Liu, Yao Chen, Khan Muhammad Kashif, Qingyuan Wang, Yongjie Liu

**Affiliations:** 1 MOE Key Laboratory of Deep Earth Science and Engineering, College of Architecture and Environment, Sichuan University, Chengdu 610065, China; liu.fl@foxmail.com (F.L.); h.zzhang@foxmail.com (H.Z.); liu.hq@foxmail.com (H.L.); chenyao1101@126.com (Y.C.); 2 Failure Mechanics and Engineering Disaster Prevention and Mitigation Key Laboratory of Sichuan Province, Sichuan University, Chengdu 610207, China; 3 Institute of Future Transport and Cities, Coventry University, CV1 5FB Coventry, UK; ac1291@coventry.ac.uk; 4 School of Architecture and Civil Engineering, Chengdu University, Chengdu 610106, China

**Keywords:** titanium alloy TC17, electron beam welded joint, welded pores, very high cycle fatigue regime, fine granular area (FGA), stress intensity factor

## Abstract

The electron beam welding process is widely used in the connection among titanium alloy material parts of aero-engines. Its mechanical properties need to meet the requirements of long life and high reliability. In this paper, the static strength and the fatigue failure behavior of the electron beam weldments of TC17 titanium alloy were investigated experimentally under low amplitude high frequency (20 kHz), and the mechanical response and failure mechanism under different external loading conditions were analyzed. In summary, the samples were found to have anisotropic microstructure. The tensile strength of the PWHT of TC17 EBW joint was ~4.5% lower than that of the base metal. Meanwhile, compared with the base metal, the fatigue strength was reduced by 45.5% at 10^9^ cycles of fatigue life. The fracture analysis showed that the fatigue failure of the welded joint of TC17 alloy was caused by the welded pores and the fatigue cracks initiated from the welded pores. A fine granular area (FGA) was observed around the crack initiation region. The existence of pores caused the stress intensity factor of the fine granular area (K_FGA_) to be inversely proportional to the fatigue life. The K_FGA_ calculation formula was modified and the fatigue crack propagation threshold of the welded joint of TC17 alloy was calculated (3.62 MPa·m^1/2^). Moreover, the influences of the effective size and the relative depth of the pores on the very long fatigue life of the electron beam welded joint of TC17 titanium alloy were discussed.

## 1. Introduction

Titanium alloys are widely used in engineering application where high strength, good fracture toughness, and resistance against fatigue fracture at ambient and elevated temperature are required to meet the safe life and damage tolerance design requirements for high-reliability conditions [1,2]. The α-β phase titanium alloy TC17 (Ti-5Al-2Sn-2Zr-4Mo-4Cr) with the β-phase stable element is mainly used in the aero-engine, compressor blade discs, and large-section forgings to save the weight and increase thrust-to-weight ratio [3,4,5]. 

The aerospace components are required to join frequently to meet the aerodynamic requirements. The welding is the most common joining method for metal structures to replace the mechanical fasteners, large forgings, casting equipment, and the complexity of machining processes. The welding process realizes the requirements for integration and weight reduction of metal components. The use of laser beam, linear friction, friction stir, and electron beam welding is continuously increasing in the aerospace components [6,7,8,9,10,11]. On the one hand, the electron beam welding (EBW) is used for welding of thick titanium plates due to its high precision, high welding speed, narrow heat-affected zone, and small deformation in welded parts [12,13]. On the other hand, the electron beam welding is carried out in a vacuum, which completely avoids the oxidation problem of titanium alloy in the atmosphere and ensures the purity of the welding seam. In conclusion, the EBW offers accurate control of process parameters which ensure the welding stability and the high welding quality thus preferred in aerospace components.

The microstructure of the electron beam welded joints differs in the heat-affected zone and the weld bead, which are observed in titanium alloys, aluminum alloys, and nickel-based alloys of welded joints [11,14,15,16]. Interestingly, despite the vacuum condition used in the welding process, the welding defects in the form of the welded pores and inclusions are still observed in the welded joints. The hydrogen content in titanium alloys aids the welded pores formation in the EBW joints [17,18]. These welded defects reduce the tensile and fatigue strength of the welded joints significantly [15,19]. The stress concentration around the welded defects promotes premature fracture of the welded joints at strength much lower than the base metal. The static and cyclic loading behavior of the welded joints varies with the size and density of the welded defects [16,20]. The mechanical behavior of the welded joints with one set of process parameters differs significantly with those welded with other parameters [18,21]. The variations in static and cyclic properties of the welded joints make their life prediction extremely challenging. The aerospace components are designed for high safety and durability. The variations in properties of the welded joints with small scale pores turn out to be the main obstacle in the qualification and certification of the EBW process in manufacturing supply chain. The aerospace components in the life cycle encounter high and low-stress amplitude fatigue cycles well beyond 10^7^ cycles. The current state of the art of the EBW joints for metallic materials is the design life using the fatigue limit up to 10^7^ cycles. However, it is now well understood that metallic materials experience fatigue fracture up to 10^10^ cycles and assuming infinite fatigue life below fatigue limit is a conservative concept [22,23]. Hence, it is now extremely important to understand the fatigue behavior of EBW joints for design life up to 10^9^ cycles. The welded pores prone characteristics of the EBW joints demand thorough investigations of the fatigue behavior.

The aero-engine components experience low cycle fatigue regime in the startup and shutdown process and very high cycle fatigue regime in the operation. The fatigue characteristics of welded joints of titanium alloy have been studied in the past, mainly focusing on the welding process and the fatigue properties in low and high cycle fatigue regime. For instance, Balasubramanian at al. [21] aimed to evaluate fatigue crack growth parameters of gas tungsten arc, electron beam and laser beam welded TC4 titanium alloy. They found that not only the threshold stress intensity factor of TC4 alloy was not influenced significantly by the welding processes but also the joint fabricated by LBW process exhibited higher fatigue crack growth resistance than EBW and GTAW joints. Jinkeun [18] compared the high cycle fatigue properties of EBW and TIG welded joints of TC4 titanium alloy and found larger defects and lower fatigue strength in EBW joints than that TIG joints. Fomin [20] focused on the effect of inherent welding-induced defects on the high-cycle fatigue behavior of laser-welded TC4 butt joints. The mechanism of FGA formation was studied and linked to the plastic zone size at the crack tip. In addition, the distribution of the pore size and position were measured and the fracture-mechanics-based framework was applied for the development of the fatigue-life estimation model. Han et al. [24] conducted that the cracks initiate from the surface of EBW joints of TC18 titanium alloy. It was found that the grain size of the welded joints decreases with an increase in the welding speed which helps in increasing the fatigue strength of the welded joints in low cycle fatigue regime. Cheng et al. [13] studied the fatigue properties of TC17/Ti60 dissimilar electron-beam-welded joints and found fatigue crack initiation from the micropores in high cycle fatigue regime. Some studies investigated the fatigue behavior of the welded joints beyond 10^7^ cycles and found a different crack initiation mechanism. Melvin [25] investigated the very long life fatigue behavior of Ti-6Al-4V welded joints. They observed the fatigue failure in the welded joints even beyond 10^7^ cycles and found a linearly decreasing S-N curve without traditional fatigue limit. Zhao et al. [16] identified that the fatigue strength of the welded joints of TC21 alloy depends on the crack initiation site. They concluded that cracks initiate from the voids and dislocation in the welded joints and the different mechanism of crack initiation from these sites produces different fatigue strength. The empirical understanding of the fatigue behavior developed from previous studies is not useful in the very high cycle fatigue regime of the welded joints. The analysis of fatigue strength variation with crack initiation sites is not sufficient for using the EBW joints in aerospace applications with very high safety and durability regulations. There is no systematic and in-depth study on the crack initiation, propagation, and failure mechanism of titanium alloy EBW joints up to very high cycle fatigue.

This study investigated the mechanical properties of TC17 EBW joints in quasi-static and cyclic loading. The fatigue life of the welded joints was investigated up to very high cycles regime by ultrasonic accelerated vibration test (20 kHz) with stress ratio R = −1. Moreover, after fatigue testing, the scanning electron microscope (SEM) was used to observe and microscopically analyze the fracture of the welded joint of TC17 alloy of fatigue specimen. In the end, the fatigue failure mechanism was investigated.

## 2. Materials and Methods

In the present study, the experiment material is TC17 which is α-β phase titanium alloy. The chemical composition of the TC17 titanium alloy is shown in Table 1. The elastic modulus is 116 GPa and the density is 4.68 g/cm^3^ at room temperature.

**Figure 1:**
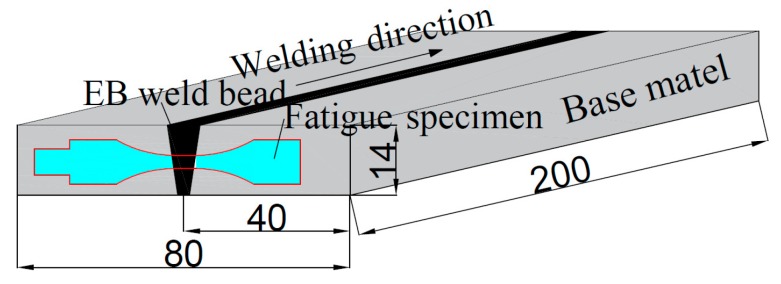
The size of the welding plate and specimen sampling (unites: mm).

**Figure 2:**
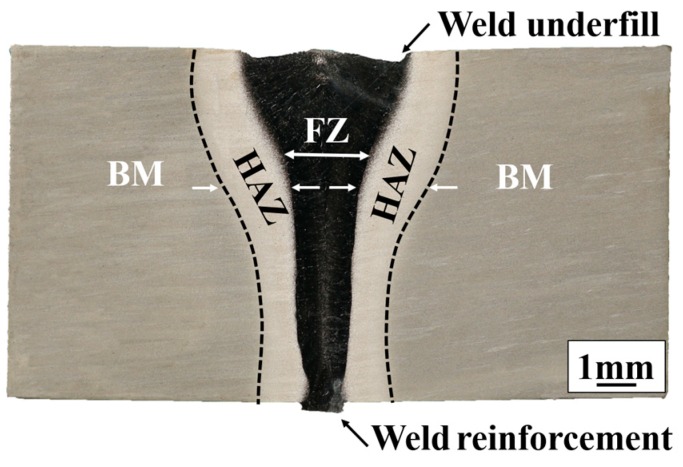
Macrostructure of electron beam welding (EBW) joint of TC17 titanium alloy.

The material was processed into test plates with a size of 200 mm × 40 mm × 14 mm by means of machining, and the welding process was performed by vacuum electron beam welding. The dimensions of the test plates are shown in Figure 1. The plate segments were carefully mechanically polished and chemically cleaned to remove oxides, oil, and moisture from the surface of the plates before welding. Electron beam welding was performed with a ZD150-C high-pressure vacuum machine (SST, Werne, Germany) with the welding direction parallel to the length of the test plates for welding. The parameters of the electron beam welding process include 150 kV accelerating voltage, 2016 mA focusing current, 10 mm/min welding speed, and 43 mA welding current. Due to the electron beam oscillation during the welding process, fish scales were formed on the surface of the welded plates. Furthermore, the electron beam welded joints of TC17 alloy were divided into a fusion zone (FZ), a heat affected zone (HAZ) and a base material zone (BM) in Figure 2. The welded joint of TC17 titanium alloy was found to have an upper and lower width of ~3 and ~1 mm, respectively. Meanwhile, the weld underfill and the weld reinforcement produced by rapid cooling can be observed in the fusion zone, as shown in Figure 2.

**Figure 3:**
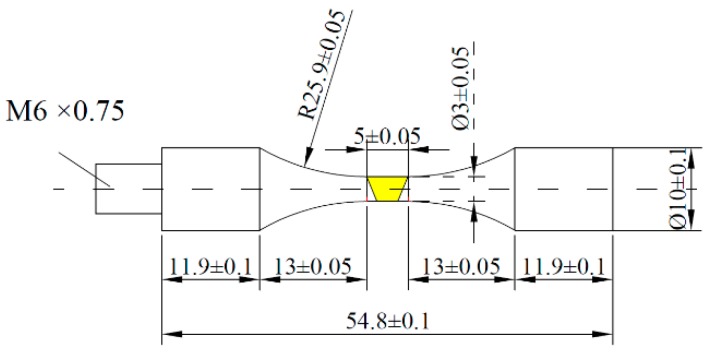
Very high cycle fatigue test specimen of EBW joints of TC17 titanium alloy (unites: mm).

Firstly, the round bar-shaped samples were extracted from the middle of the welded plates and perpendicular to the weld direction in Figure 1. Milling of the surfaces of the samples was conducted to get rid of geometrical defects after electron beam welding, such as the welded reinforcement and welded underfill which play a role of stress concentrators and have a strong weakening effect on the fatigue life. Secondly, the samples were post-weld heat treated (PWHT) of 630 °C for 2 h in air cooling [15] to remove the welded residual stresses. Lastly, the round bar-shaped samples were processed into very high cycle fatigue specimens after heat treatment. The specimen geometry was designed to a smooth cylindrical (dog-bone) shaped fatigue specimen, ensuring that the fusion zone, the heat-affected zone, and the base metal with the same external loading and meeting the resonance requirement of 20 kHz. The calculated dimensions of the specimen are shown in Figure 3. Before the experiment, the middle parallel section, and the arc over the section of the fatigue specimen were polished to the mirror state and corroded with Keller’s reagent (10 mL HF+ 15 mL HNO_3_ + 75 mL H_2_O).

The tensile tests of TC17 EBW joints were performed with a vertical electronic universal testing machine (SHIMADZU AGS-X, Kyoto, Japan). The fatigue tests were carried out on USF-2000 very high cycle fatigue testing machine (SHIMADZU, Kyoto, Japan) at an ultrasonic frequency of 20 kHz. The stress ratio R = -1 was used in testing and the surface of the specimen was cooled using cooling air. Additionally, the microstructure of the welded joints was observed by JSM-6510LV scanning electron microscope (SEM, JEOL, Tokyo, Japan). The morphology of the fatigue fractures after the very high cycle fatigue test was observed and analyzed.

## 3. Results

### 3.1. Micrograph of Welded Joint of TC17 Titanium Alloy

**Figure 4:**
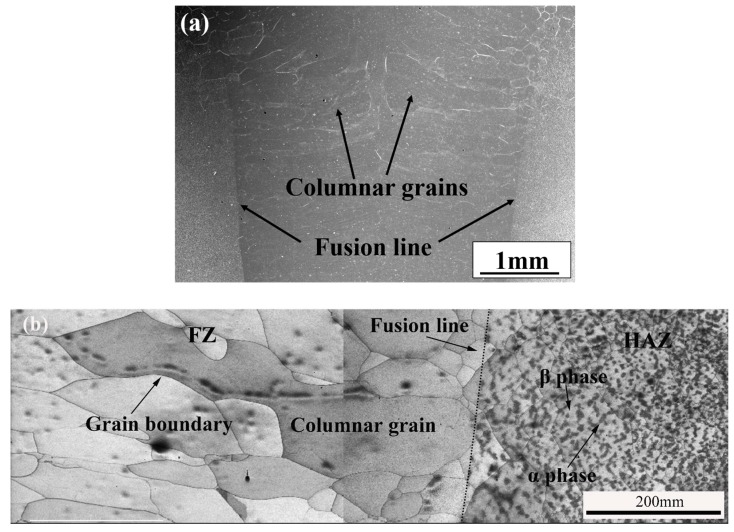
Micrograph of the as-welded TC17 EBW joint, (**a**) the welded zone, (**b**) the fusion zone and the heat affected zone.

Figure 4a shows the micrograph of as-welded TC17 EBW joint. The coarse columnar grains in the fusion zone can be clearly observed from the figure. There are obvious fusion lines between the fusion zone and the heat affected zone. The columnar crystal growth was observed perpendicular to the centerline of the fusion zone. Figure 4b shows the fusion zone and the heat-affected zone of the TC17 EBW joint. The coarse columnar crystals and grain boundaries of the fusion zone can be clearly observed. Moreover, the average grain sizes were from 200 to 500 μm. A large amount of α-phase is distributed in the heat-affected zone, meanwhile, the grain size of the heat affected zone is much smaller than the size of the fusion size of the TC17 EBW joint.

### 3.2. Tensile Properties and Fracture of TC17 Alloy EBW Joints

**Figure 5:**
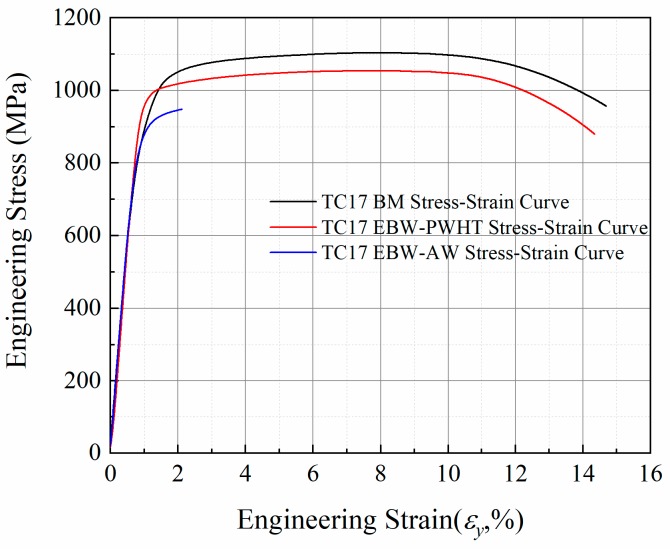
Stress-strain curves of the base metal, as-welded TC17 EBW joint, and PWHT of TC17 EBW joint.

**Figure 6:**
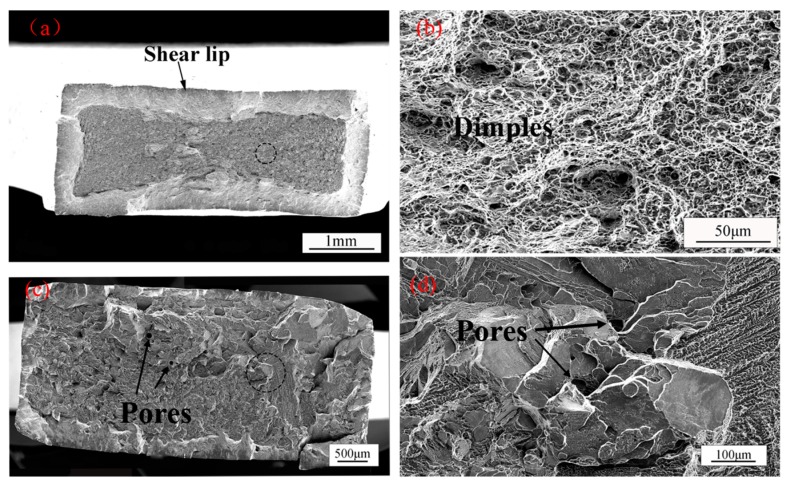
The tensile fractures of TC17 alloy EBW joints, (**a**) BM of the fracture of PWHT TC17 EBW joint, (**b**) BM of the fracture of PWHT TC17 EBW joint enlarged view, (**c**) FZ of as-welded TC17 EBW joint fracture, (**d**) FZ of as-welded TC17 EBW joint fracture part enlarged view.

The stress-strain curves of the base metal, as-welded (AW), and PWHT of TC17 welded joints are shown in Figure 5. The room temperature tensile test data of TC17 EBW joints is shown in Table 2. The strength of the base metal is higher than those of as-welded joints and PWHT joints (Table 2). The tensile strength of the as-welded TC17 EBW joints is about 948 MPa under nominal stress that is almost 86% of the tensile strength of the base metal (1103 MPa). The as-welded joints show almost no ductility and fail just beyond the yield strength. However, the as-welded joints restored the strength and ductility of the joints by PWHT. The tensile strength of the welded joint of TC17 titanium alloy by post-weld heat treatment is about 1053 MPa, which means nearly about 95.5% of the strength of the base metal, and the elongation of fracture is about 17%. Compared with the as-welded TC17 EBW joint, the tensile strength is increased by about 10% of TC17 EBW joint by PWHT. The as-welded joints fractured from the fusion zone. Nevertheless, the PWHT welded joints fractured from base metal. This showed that PWHT was effective in increasing the strength of the fusion zone. Since the heat treatment released the internal stress generated by the welding and caused a transition between α phases and β phases in the FZ and the HAZ. During the heat treatment process, supersaturated solid solution β phases with martensitic α’ phases were transformed into secondary α lamella embedded in the β matrix, which could enhance the mechanical behavior of TC17 EBW joint [15]. Fracture observation showed that the PWHT of TC17 alloy EBW joint undergone ductile fracture under tensile action, which has an obvious shear lip in Figure 6a. In addition, a large number of dimples are distributed on the final failure fracture in Figure 6b. Nevertheless, the as-welded TC17 EBW joint undergone brittle fracture under tensile action. Figure 6c,d showed the fracture surface of as-welded specimens is brittle fracture with cleavage planes. Besides, the welded pores on the surface fracture can be observed. 

### 3.3. S-N Curve of Electron Beam Welded Joints of TC17 Titanium Alloy by PWHT

**Figure 7:**
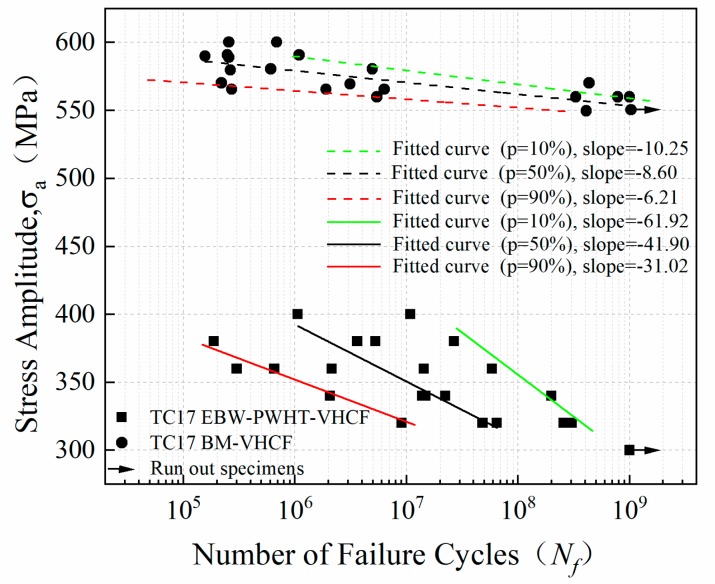
S-N curves of the welded joints of TC17 alloy in very high cycle fatigue regime.

Figure 7 shows the S-N curves of the welded joints with PWHT and BM of TC17 titanium alloy. The data for runout specimens at 10^9^ cycles is marked with an arrow (Figure 7). The results show that the S-N curves of the welded joints are linearly decreasing with an increase in the number of cycles. The fatigue failure is observed even beyond 10^7^ cycles which shows that the traditional fatigue limit is not there. As the fatigue life of welded joints ranges from 1.88 × 10^5^ to 1.00 × 10^9^ cycles, the stress level ranges from 400 MPa to 300 MPa. The S-N curves of the specimens show large scatter as compared to the base metal S-N curves. For fatigue strength design, P-S-N curves obtained for materials with different failure probabilities are more applicable. P-S-N curves effectively solve the uncertainty introduced by the dispersion of fatigue life of materials and can be used as a more intuitive basis for fatigue strength design. Moreover, the Basquin stress-fatigue relationship was used for the fatigue life prediction of welded joints. The relationship Δ*σ* = 511.72(2N*_f_*)^−0.022^ was obtained from the fitting of S-N curves, where Δσ is the stress amplitude of test loading, and N*_f_* is the fatigue life of TC17 EBW joints.

For comparison, the fatigue results of the base metal are presented in Figure 7. The difference in the fatigue strength of the base metal and the welded joints at 10^9^ cycles is ~250 MPa. It can be seen that the fatigue strength of the welded joints is significantly lower than the same for the base metal. In other words, the fatigue strength of the welded joint is only 54.5% of the fatigue strength of the base metal at 10^9^ cycles. However, the difference in the ultimate tensile strength of the base metal and the welded joints is only ~100 MPa. Meanwhile, the results are consistent with other studies on different titanium alloy materials of TC4 [21,25,26] and TC21 [16,27]. The higher difference in the fatigue strength for the welded joints as compared to the base metal show that cyclic loading is more detrimental to the strength of the welded joints. This may be attributed to the welded defects in the welded joints which will be discussed later in detail. The higher difference in the fatigue strength of the welded joints than base metal at 10^9^ cycles as compared to the fatigue strength at 10^5^ cycles shows that the effects of the welded defects on fatigue life reduction are higher in the very high cycle fatigue regime. It can be said that the design of EBW joints for longer fatigue life will need a careful investigation of the fatigue strength up to very high cycles.

### 3.4. Fatigue Fracture Analysis of Welded Joint of TC17 Alloy

**Figure 8:**
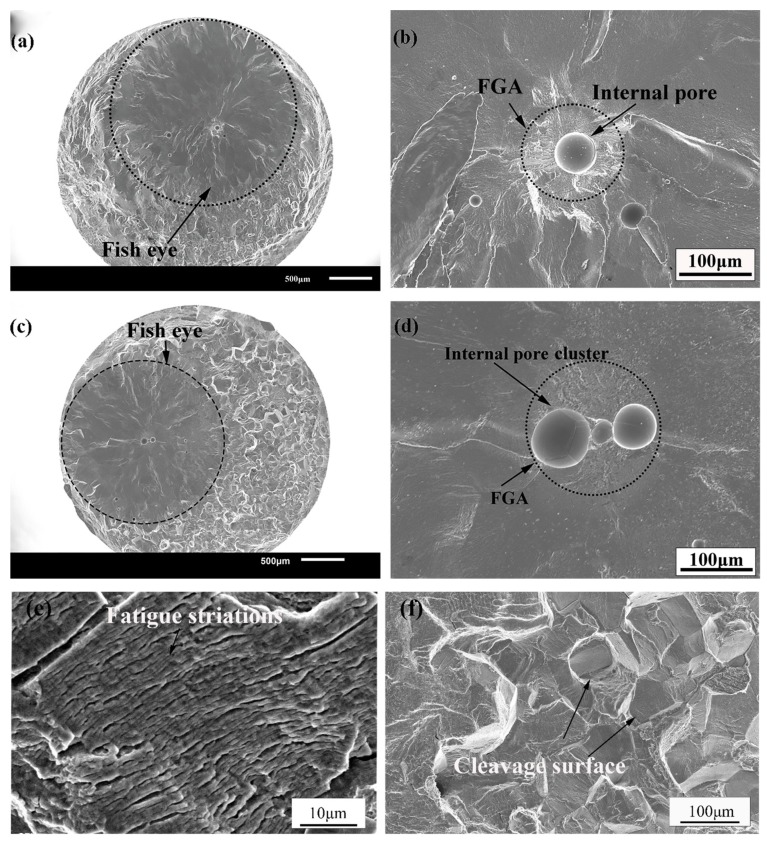
Micrographs of fatigue fracture of the welded joint of TC17 titanium alloy, (**a**) overall shape of a single pore of fatigue fracture, (**b**) morphology of internal single pore fatigue initiation area $\left(\sigma_{a}=400\;\mathrm{MPa},N_{f}=1.0860\times 10^{7}\right)$, (**c**) overall pore morphology of fatigue fracture, (**d**) internal pore cluster of fatigue initiation area $\left(\sigma_{a}=360\;\mathrm{MPa},N_{f}=2.1414\times 10^{6}\right)$, (**e**) fatigue striations of fatigue fracture, (**f**) cleavage surface of the TC17 EBW joint.

**Figure 9:**
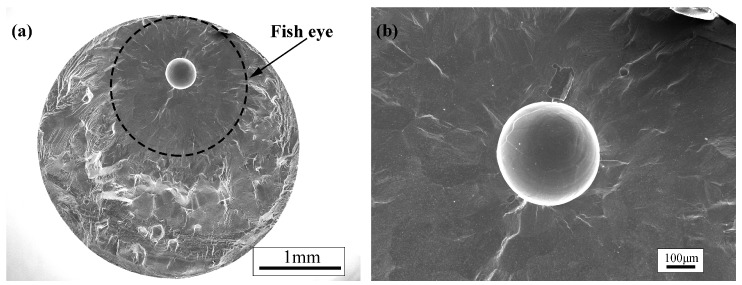
Micrographs of the bigger pore fatigue fracture of TC17 titanium alloy welded joint, (**a**) overall shape of the welded joint fracture, (**b**) fatigue source area morphology of the internal pore of the welded joint $\left(\sigma_{a}=340\;\mathrm{MPa},N_{f}=2.0606\times 10^{6}\right)$.

The fracture surfaces of the fatigue specimens were observed by the scanning electron microscope (SEM). The fracture locations of the fatigue specimens are distributed in the fusion zone and the heat-affected zone of welded joints of TC17 alloy regardless of the high-stress amplitude or the low-stress amplitude that is mainly due to the fusion zone. The reason is the fusion zone undergoes rapid melting and grain growth, resulting in coarse grains, and it is easy to produce the welded pores in the fusion zone during the electron beam welding process [17]. The fatigue crack initiation was observed from the welded pores distributed in the internal, subsurface and surface of the TC17 EBW joints. There are obvious characteristics of crack initiation zone, crack propagation zone and final failure zone on the fracture of the welded joints. Figure 8 shows the fracture surfaces of the welded joints loaded with 400 MPa and 360 MPa. It can be clearly observed the fracture surface has a "fisheye" region around the pore from where the crack initiation took place, as shown by the dotted circle in Figure 8a,c. The fracture surface is smooth and flat, forming a radial decorative pattern centered on the pores. A distinct fine granular area (FGA) called by Sakai et al. [28] and fracture caused by transgranular crack propagation were observed around the pores in the "fisheye" region. The section is rough with high-density cleavage planes outside the "fisheye" region, as shown in Figure 8f. Under the high-power electron microscope, the fatigue striations could be observed in the crack propagation zone perpendicular to the crack propagation direction, as shown in Figure 8e. This shows a high number of fatigue cycles consumed in the crack initiation and early propagation, even reaching the majority of the total fatigue life [22,29,30]. The upper and lower crack faces are pressed against each other to form a smooth and flat "fisheye" region, and later the fatigue cracks propagated rapidly forming rough cleavage planes [22]. In some cases, the crack initiation was observed from the cluster of multiple pores. The FGA from the cluster of multiple pores is similar to those with single pore, as shown in Figure 8b. Moreover, it was found that when there is the pore cluster in the initiation zone, the fatigue life is still reduced even under low-stress amplitude. It can be said that the increase in the number of pores results in a larger area of stress concentration to reduce the fatigue life. Multiple short cracks around the pores emanate and coalescence to form long cracks reducing the fatigue life of the welded joints. Figure 9 shows the fracture surface of specimen loaded with 340 MPa stress. The fracture surface of specimen shows a fisheye without FGA region around a large pore size (200 μm diameter). In other words, the fatigue cracks stride over the initiation zone and directly enter the early propagation area. It is worth noting that for the non-metallic inclusions in the welded joints of high-strength steel, it was found that the size and position of non-metallic inclusions are closely related to the fatigue life of the material. Meanwhile, a new "Z" parameter fatigue life prediction model is proposed based on the Murakami model, which is related to the local stress concentration and the defects size and location [19,31]. Therefore, the number of pores, the size of pores and the location of pores in the initiation zone will play a crucial role in the very long fatigue life of the welded joint. The mechanism of the influence of the welded pores for the fatigue life will be described in detail in the following sections.

## 4. Discussion

### 4.1. Relationship between Static Strength and Fatigue Strength of TC17 Welded Joints

Minakawa [32] summarized the relationship between tensile strength and fatigue strength of TC4 alloy. The fatigue strength of the material increases with the increase of tensile strength. However, when the tensile strength reaches 1000 MPa or more, it is increased and the fatigue strength is no longer increased at R = −1. Because fatigue crack initiation is mainly caused by grain slip, and it is known that the yield strength of the material is directly related to the critical shear stress of the grain slip. Therefore, the yield strength is considered to be related to the fatigue strength [33]. Murakami [34] showed that the fatigue strength of the material is not the threshold stress of crack initiation and the cracks do not propagate until the threshold stress acting on the material. Besides, there is dispersed distribution of the pores in the welded joints of TC17 titanium alloy. The effects of the pores on the quasi-static properties of the welded joint are not significant (see Figure 5), and they are mainly affected by the grain morphology of the welded joint (Figure 6c). However, the fatigue properties of the welded joint are seriously affected by the welded pores. It has been proved that the very high cycle fatigue strength of the welded joints is only 30% of the tensile strength of the welded joints (Figure 5 and Figure 7). Hence, the static and fatigue strength can be approximately correlated as:

S*_f_*_(tension)_= (0.3~0.45) S_u_ (1)

where S_u_ represents the ultimate tensile strength of the welded joint of TC17 alloy, and S*_f_*_(tension)_ represents the fatigue limit of the welded joint of TC17 alloy.

### 4.2. Study on the Threshold Value of very High Cycle Fatigue Fracture

In very high cycle fatigue regime, the life of fatigue crack initiation accounts for almost 90% of the total fatigue life [30,35,36,37,38]. The crack initiation is very sensitive to the local inhomogeneity in the microstructure of the material. Studies show that the fatigue failure induced by the internal initiation of TC17 alloy is caused by the slip fracture of the primary α phases [5]. In the forging process, the base material of TC17 alloy may form the inhomogeneous microstructure size and distribution including local strong texture. Under the cyclic loading, the local microscopic strain distribution of the material may be affected which may initiate the cracks [5]. In addition, the internal cracks are inhibited and hindered by the internal vacuum environment during the short crack propagation stage [39] resulting in relatively long fatigue life of internal crack initiation under the same cyclic stress loading. Nevertheless, the weld bead passes through the gradient from melting temperature to room temperature in the welding process. The material in the welding process shrinks but is limited by the lower temperature plate. The fusion zone, in particular, was found to have higher geometric discontinuity than another two zones, leading to easily fatigue fracture of the welded joint here. The crack initiation from the pores develops a rough region (FGA) in the vicinity. When the crack tip stress intensity factor of the FGA reaches the crack propagation threshold of the material, the cracks enter the steady-state propagation stage.

Generally, high-strength steel in the very high cycle regime fatigue test, a fine granular area (FGA) is formed near the inclusions inside the material [28]. FGA is the crack initiation zone, and the fatigue life of FGA is the crack initiation life [36]. In addition, it has been confirmed that the fatigue life of FGA accounts for the large part of the total fatigue life [22,23,36,37]. The fracture of the welded joint indicates that the crack originates from the welded pores. In short, FGA is formed around the small pores, then the crack enters the stable propagation stage. While the FGA is not observed around the larger pore, the crack enters directly in the early propagation stage from the pores reducing the fatigue life of TC17 EBW joints. Therefore, the crack propagation threshold determines whether FGA is formed around the pores in the initiation zone and the stress intensity factor of the pores is greater than that of FGA which is not formed around the pore.

In the very high cycle fatigue regime, the stress intensity factor K is often used as the characterization for describing crack initiation and propagation. In this study, the maximum K_M_ expression of the stress intensity factor of the surface or internal defect initiation crack of the sample was estimated based on the analysis results of Murakami [34,39]. When cracks initiate from surface defects:

(2) $$K_{M}=0.65\Delta \sigma (\pi \sqrt{area_{\max}})$$

**Figure 10:**
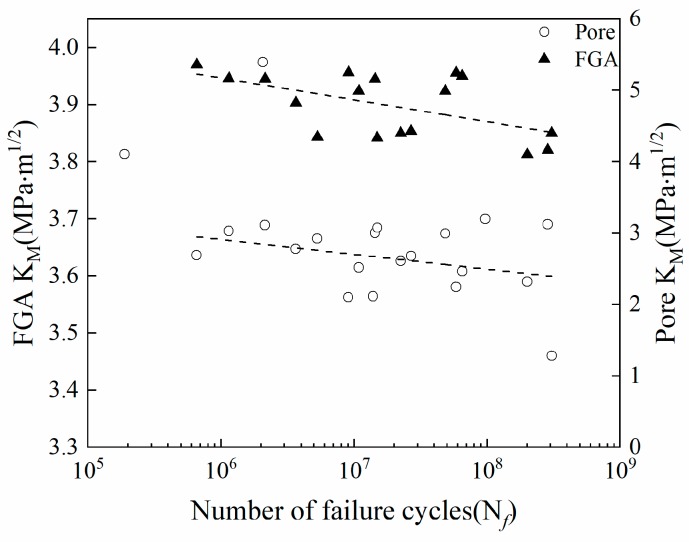
Relationship between K_M_ of pores and fine granular area (FGA) and fatigue life.

When cracks initiate from subsurface or internal defects:

(3) $$K_{M}=0.5\Delta \sigma (\pi \sqrt{area_{\max}})$$

where ∆σ is the stress range and area_max_ is the maximum projected area of the defect perpendicular to the plane of the tensile stress. In the tensile-pressure fatigue cycle with a stress ratio of −1, only the tensile stress acts on the fatigue crack initiation and propagation, so stress amplitude σ_a_ is used instead of ∆σ in calculating the initial stress intensity factor. Meanwhile, when there is the number of pores in the fatigue initiation region, the sum of the pore cluster areas is considered to calculate the stress intensity factor of the pore cluster, and the calculation result is shown in Figure 10.

The stress intensity factor amplitudes K_M_ of FGA are distributed between 3.81 and 3.96 MPa·m^1/2^ (Figure 10). The stress intensity factors of FGA decrease with an increase in the fatigue cycles of the welded joint. However, it has been found in earlier studies [23,28,36] that the K_FGA_ is a constant independent of the fatigue life which is related to the threshold value of the crack propagation. It can be found that the stress intensity factor corresponding to the pores originating from the crack origin of the welded joint decreases with an increase in the fatigue cycles. Whereas, the pore clusters are distributed in the FGA, as shown in Figure 8. A linear fit was performed on the two sets of data, and it was found that the two fitted straight lines were approximately parallel, showing the same downward trend. Therefore, it can be said that the pores change in K_FGA_ resulting in the inverse relationship between K_FGA_ and the fatigue life of the welded joint. The conventional method for calculating the FGA stress intensity factor is not suitable for the welded joint due to the pores. In order to avoid the influence of the pores on the stress intensity factor of FGA, Equations (2) and (3) are revised, and the modified formulas are proposed for calculation of the effective FGA stress intensity factor of the welded joint. When crack initiation occurs from the surface pores:

(4) $$K_{\mathrm{FGA}}=0.65\Delta \sigma (\pi \sqrt{\left(area_{FGA}{}_{\max}-area_{pore\max}\right)}$$

**Figure 11:**
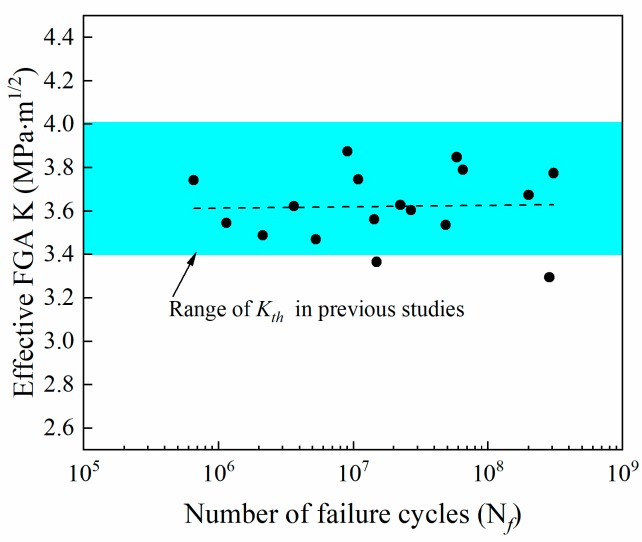
Relationship between effective stress intensity factors of FGA (K_FGA_) and fatigue life.

When crack initiation occurs from the subsurface or internal pores:

(5) $$K_{\mathrm{FGA}}=0.5\Delta \sigma (\pi \sqrt{\left(area_{FGA}{}_{\max}-area_{pore\max}\right)}$$

where $area_{FGA}{}_{\max}$ is the maximum projected area of the FGA perpendicular to the tensile stress plane, $area_{pore\max}$ is the sum of the maximum projected areas of the respective pores perpendicular to the tensile stress planes. The variation in the effective FGA stress intensity factor with fatigue cycles to failure is shown in Figure 11. By linearly fitting the calculation results, it can be seen that the effective FGA stress intensity factor of K_FGA_ does not change with the number of cyclic loadings and remains substantially constant. For titanium alloys, the crack initiation threshold (K_th_) obtained from many previous experimental results [36,40,41] is 3.4~4.0 MPa·m^1/2^. Considering the measurement error and calculation accuracy, the average value of K_FGA_ obtained by this work is 3.62 MPa·m ^1/2^ which is included in the crack propagation threshold of titanium alloy. Therefore, the stress intensity factors of K_FGA_ are the effective threshold for fatigue crack propagation of welded joints of TC17 titanium alloy. In the current study, similar fine granular areas are found in special areas with high fatigue life, and the stress intensity factor of K_M_ is also close to K_th_. This characteristic area has intrinsic existence during very high cycle fatigue regime.

### 4.3. Effect of Welded Pores on very High Cycle Fatigue Properties of Welded Joints

In the very high cycle regime fatigue state of high-strength steel, it is difficult to form a slip band on the non-uniform microstructure of the sample surface due to the low cyclic stress. The fatigue crack initiation site shifts surface to the subsurface of the specimen. Under the action of the very long cyclic loading, the local stress concentration around the inclusions is caused by a mismatch of properties between the inclusions and the matrix, which is the main inducing factor for the formation of fatigue cracks [38]. The inclusions in the subsurface of material are separated from the matrix due to the increase in strain and dislocation density [31]. In cycle loading, the detachment occurs at the interface resulting in microcracks. In conclusion, the fatigue cracks thus expand and form a relatively rough FGA around the inclusions [34,38]. Nevertheless, the pores are generated by the welding of the TC17 EBW joints. The fatigue crack initiation mechanism of the welded joint is different from that of TC17 base metal, but it has a certain similarity with the fatigue crack initiation mechanism of high strength steel. The existence of the pores causes the geometric conditions inside the specimen to be discontinuous. Under the cyclic loadings, stress concentration occurs around the pores, so that the stress around the pores is much larger than the loading stress [38], resulting in the local plastic strain being gradually accumulated, and the slip band is easier to form around the pores, and then initiates fatigue microcracks. The microcracks repeatedly open and close, which causes early fatigue damage. When the microcracks reach a certain size, the fatigue cracks enter the early propagation stage to form the fisheye (Figure 8 and Figure 9). Besides, the macroscopic cracks are formed, then the cracks enter the rapid propagation stage up to the final failure of the material.

**Figure 12:**
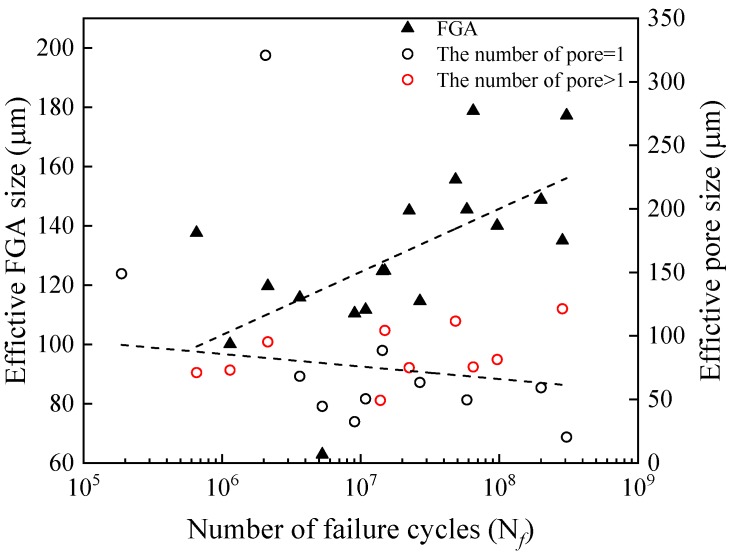
Relationship between the effective sizes of pores and FGA and fatigue life.

The above discussion shows that the pores affect the calculation of K_FGA_, and make the original K_FGA_ in the initiation zone correlate with fatigue life to a certain extent. Therefore, the pores affect the stress-life relationship of welded TC17 alloy joints, and the data show greater dispersion (Figure 7). In this paper, the effective size of pores, the relative depth of pores and the number of the pores in the initiation zone of the welded joint specimen are counted. Figure 12 is a diagram of the relationship between the effective sizes of pores and FGA and fatigue life in the fracture initiation zone of welded TC17 alloy joint. The effective sizes of the pores were calculated from the square root of the total area of the pores in the initiation zone. The effective sizes of FGA were calculated by the square root of the effective FGA area. The red ring indicates that the number of pores in the fatigue crack initiation zone is greater than 1. However, the black ring indicates the number of the pores in the fatigue crack initiation zone is 1. When the number of cyclic loadings is less than 10^7^ cycles, the size distribution of effective FGA is dispersed, which shows the sizes of effective FGA are mainly affected by higher stress level; when the number of cyclic loadings is more than 10^7^ cycles, that at lower stress level, the sizes of effective FGA increase with the increase of fatigue life, and their sizes range from 100 to 160 μm. For the pore cluster, with the increase of the number of cyclic loadings for the welded joints of TC17 alloy, the effective sizes of pores decrease slightly. Most of the effective sizes of pores are between 30 and 150 μm. The larger pores in the specimen show that there is a lower fatigue life of welded joints, even though under low-stress amplitude. The stress intensity factors of the pores are larger than that of K_FGA_ due to large pores (Figure 10). The fatigue cracks skip the formation process of FGA, resulting in the absence of morphological characteristics of FGA. The stress concentration around the pores increases the stress forming the plastic slip bands leading to the crack initiation [38]. Generally, the fatigue cracks initiate from the surface, mainly in the low and high cycle fatigue regime. Therefore, it can be inferred that the fatigue life of the fatigue crack initiation on the inner surface of the pores is much shorter than that of the internal FGA mode. After a short process of cracking initiated by surface slip band, the fatigue crack propagation stage is directly entered, resulting in final failure. To the extent that the effective size of pores seriously restricts the fatigue life of welded joints of TC17 alloy.

**Figure 13:**
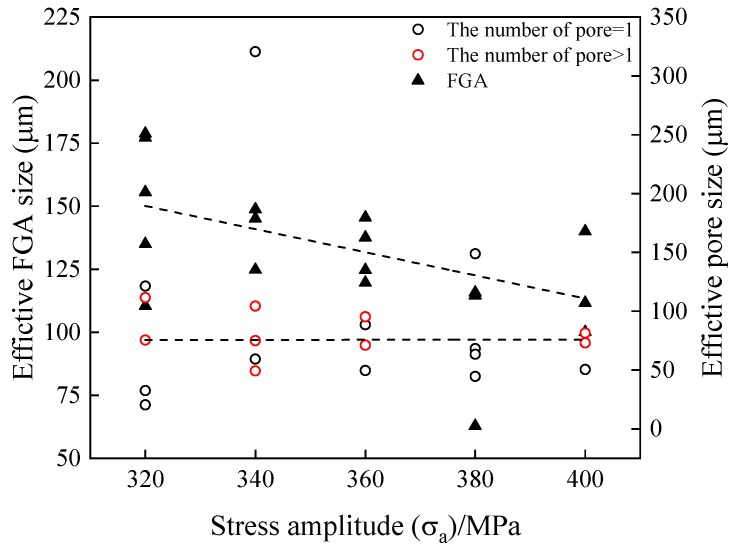
Relationship between the effective sizes of the pores and FGA and stress amplitudes.

Figure 13 shows the variation of the effective sizes of pores and FGA with stress amplitude in welded joints. It can be seen from the figure that the effective sizes of FGA decrease with an increase in the loading stress. However, the effective sizes of the pores leading to the crack origin basically are constant with the change in stress amplitude. The effective sizes of the FGA were found higher for lower stress amplitude. The fatigue life of specimens with larger FGA was found to be higher. To conclude, it can be said that a higher number of fatigue cycles are required to increase the FGA size. This phenomenon was also observed by Sakai et al. [26,29] in SUJ2 steel.

**Figure 14:**
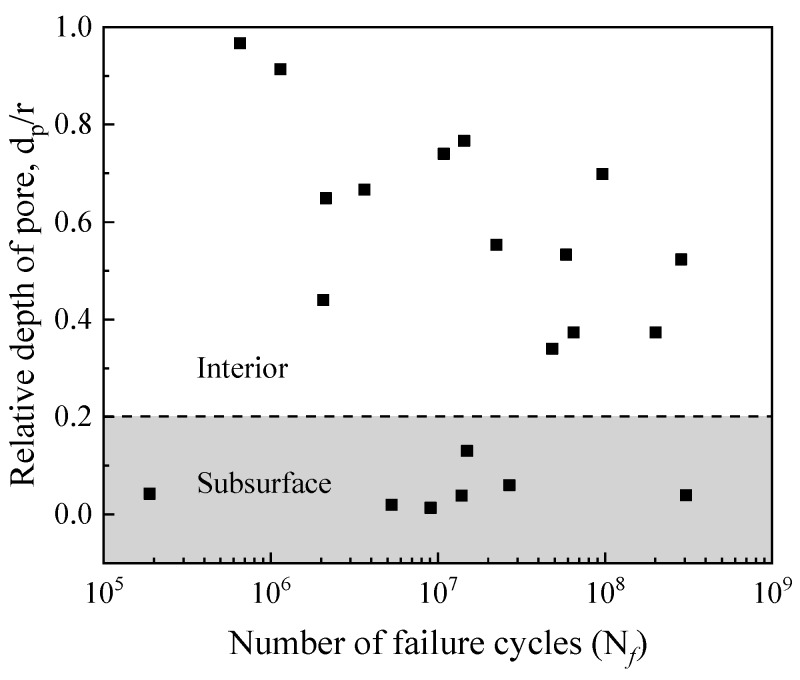
Relationship between relative depths of the pores and fatigue life.

**Figure 15:**
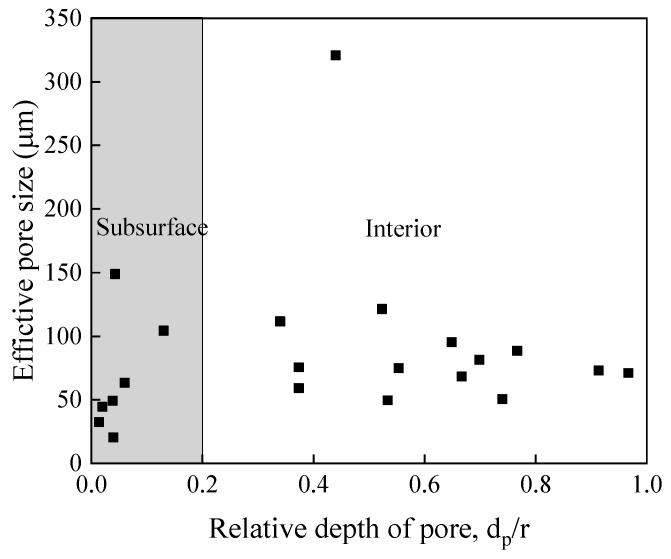
Relationship between pores sizes and relative pores depths.

Figure 14 shows the variation in the relative depths of the pores (*d_p_/r*) [31] with the fatigue life of the specimen. Here *d_p_* is the closest distance between the center of the pores and the surface of the specimen. The *r* is the radius of the specimen. The fatigue life of the specimen was found to be increasing with the decrease in the relative depths of pores. The pores are randomly distributed in the specimens and there is no relationship between the relative depths of pores and their sizes, as shown in Figure 15. In summary, when the number of cyclic loadings is less than 10^7^ cycles (high or low cycles regime), the higher loading stress level determines the fatigue life of welded joints, and the influence of pores on the fatigue life of welded joints is small. When the number of cyclic loadings is greater than 10^7^ cycles, the sizes of effective FGA around the pores increase. Meanwhile, the effective sizes and the relative depths of the pores determine the very long fatigue life of welded joints. When the stress intensity factor of FGA reaches ~3.4–3.6 MPa·m^1/2^, the cracks propagate leading to failure.

## 5. Conclusions

(1) On one hand, the tensile strength of as-welded TC17 alloy welded joint is around 86% of the base metal and the fracture occurs in the fusion zone while it has almost no ductility. On the other hand, the tensile strength of the welded joint of TC17 alloy by PWHT is about 95.5% of the base metal, and the fracture occurs in the base metal with a fracture elongation of 17%. Furthermore, the welded pores were observed on the surface of the as-welded TC17 EBW fracture. In short, this shows that the existence of pores does not obviously affect the static mechanical properties of the welded joint.
(2) In the ultrasonic accelerated vibration fatigue test with stress ratio R = -1, the fatigue S-N curve of welded TC17 alloy joints shows a continuous downward trend. There is no fatigue limit at 10^7^ cycles. Nevertheless, at 10^9^ cycles, the fatigue strength of welded joints is about 300 MPa, and the fatigue strength of the welded joints is only 54.5% of the base metal. In conclusion, the fatigue strength of welded joints is far lower than that of base metal;
(3) The fatigue fracture locations of welded TC17 alloy joints for specimens are mainly in the fusion zone and the heat-affected zone. In addition, the pores inside the specimen become the source of fatigue crack initiation which has an obvious fisheye region consisting of FGA. Moreover, the effective sizes and the depths of the pores jointly determine the very long fatigue life of the welded joint.
(4) When the fatigue crack initiation occurs in the inner or subsurface of the specimen, there is the FGA in close proximity of the pores and the smooth area around them. Afterward, the calculation formula of K_FGA_ is modified and the average effective K_FGA_ value is 3.62 MPa.m^1/2^. To conclude, the value has nothing to do with the fatigue life of the welded joint and is considered as the threshold value of stable crack growth.

## Figures and Tables

**Table 1:** TC17 (Ti-5Al-2Sn-2Zr-4Mo-4Cr ) titanium alloy chemical composition (wt %).

Al	Sn	Zr	Mo	Cr	Ti
4.5–5.5	1.6–2.4	1.6–2.4	3.5–4.5	3.5–4.5	80.7–85.3

**Table 2:** Tensile test data of TC17 EBW joints at room temperature.

Materials	Ultimate Tensile Strength, MPa; (*S*)	Yield Strength, MPa; (*S*)	Elongation, %; (*S*)
TC17 BM	1103 (20.0)	996 (12.8)	18 (0.45)
TC17 EBW-AW	948 (11.6)	869 (13.5)	5 (0.76)
TC17 EBW-PWHT	1053 (32.7)	980 (11.5)	17 (0.36)

*S* represents the standard deviation.
